# Influenza A Virus Nucleoprotein Exploits Hsp40 to Inhibit PKR Activation

**DOI:** 10.1371/journal.pone.0020215

**Published:** 2011-06-15

**Authors:** Kulbhushan Sharma, Shashank Tripathi, Priya Ranjan, Purnima Kumar, Rebecca Garten, Varough Deyde, Jacqueline M. Katz, Nancy J. Cox, Renu B. Lal, Suryaprakash Sambhara, Sunil K. Lal

**Affiliations:** 1 Virology Group, International Centre for Genetic Engineering & Biotechnology, New Delhi, India; 2 Influenza Division, National Center for Immunization and Respiratory Diseases, Centers for Disease Control and Prevention, Atlanta, Georgia, United States of America; University of Georgia, United States of America

## Abstract

**Background:**

Double-stranded RNA dependent protein kinase (PKR) is a key regulator of the anti-viral innate immune response in mammalian cells. PKR activity is regulated by a 58 kilo Dalton cellular inhibitor (P58^IPK^), which is present in inactive state as a complex with Hsp40 under normal conditions. In case of influenza A virus (IAV) infection, P58^IPK^ is known to dissociate from Hsp40 and inhibit PKR activation. However the influenza virus component responsible for PKR inhibition through P58^IPK^ activation was hitherto unknown.

**Principal Findings:**

Human heat shock 40 protein (Hsp40) was identified as an interacting partner of Influenza A virus nucleoprotein (IAV NP) using a yeast two-hybrid screen. This interaction was confirmed by co-immunoprecipitation studies from mammalian cells transfected with IAV NP expressing plasmid. Further, the IAV NP-Hsp40 interaction was validated in mammalian cells infected with various seasonal and pandemic strains of influenza viruses. Cellular localization studies showed that NP and Hsp40 co-localize primarily in the nucleus. During IAV infection in mammalian cells, expression of NP coincided with the dissociation of P58^IPK^ from Hsp40 and decrease PKR phosphorylation. We observed that, plasmid based expression of NP in mammalian cells leads to decrease in PKR phosphorylation. Furthermore, inhibition of NP expression during influenza virus replication led to PKR activation and concomitant increase in eIF2α phosphorylation. Inhibition of NP expression also led to reduced IRF3 phosphorylation, enhanced IFN β production and concomitant reduction of virus replication. Taken together our data suggest that NP is the viral factor responsible for P58^IPK^ activation and subsequent inhibition of PKR-mediated host response during IAV infection.

**Significance:**

Our findings demonstrate a novel role of IAV NP in inhibiting PKR-mediated anti-viral host response and help us understand P58^IPK^ mediated inhibition of PKR activity during IAV infection.

## Introduction

Influenza A viruses (IAV) are negative sense segmented RNA genome viruses [1], [2] which can rapidly develop resistance to the drugs available against them [3]. These viruses pose a continuing threat of pandemics, thus it is imperative to develop novel strategies to prevent their infection and spread [4]. Interactions between viral proteins and host factors are often crucial for successful replication of the virus in host cells [5]. Many of these interactions are aimed at overcoming the early innate immune response of infected cells against the virus [6]. Mammalian cells respond to viral infections through several innate immune mechanisms [7]. One such crucial antiviral mechanism is activation of PKR (a dsRNA dependent protein kinase) which is phosphorylated upon encountering viral dsRNA [8]. Activated PKR has several downstream substrates, one of which is the eukaryotic translation initiation factor 2 alpha subunit (eIF2α) [9]–[11]. Phosphorylation of eIF2α by activated PKR renders it unable to participate in translation initiation leading to translation arrest and inhibition of protein synthesis from viral mRNAs [12], [13]. Another effector function of PKR is activation of transcription factor IRF3, which leads to the expression of IFN β and inhibition of virus replication [14], [15]. Being such a crucial component of the host innate immune system, PKR is tightly regulated by cellular inhibitors [16] and very often targeted by viral proteins [17]–[20]. For example, the non-structural protein 1 (NS1) of influenza virus directly binds to PKR and prevents its activation [21], [22]. Apart from PKR inhibition, NS1 is also involved in the inhibition of other cellular signaling cascades, which lead to the activation of anti-viral interferon response [23], [24]. PKR activity is also inhibited by a cellular 58 kDa protein, P58^IPK^, which promotes influenza viral replication [25], [26]. In naïve cells, P58^IPK^ exists in an inactive state in a complex with heat shock protein 40 (Hsp40), which becomes active upon release from this complex [27]. Influenza virus infection leads to the dissociation of P58^IPK^ from Hsp40 and suppression of the PKR response [28], [29]. However, neither the viral component nor the mechanism responsible for this event is known to-date.

Segment 5 of the influenza virus genome encodes for 498 amino acids Nucleoprotein (NP) whose primary function is viral genome encapsidation [30]. Apart from that, NP is also known to interact with several viral and host factors and play additional roles in the viral life cycle [31]–[37]. We were interested in identifying new cellular interactors of NP from a highly virulent A/H5N1 bird-flu isolate {A/Hatay/2004(H5N1)}, which may facilitate viral replication. For this a H5N1 NP was used as bait to search for novel interactors in a yeast two-hybrid system based screen of human lung cDNA library. In the screen, we identified that IAV NP interacts with human chaperone heat shock protein 40 (Hsp40) [38]. Considering the known role of Hsp40 in regulation of PKR activity during influenza A virus infection [27], we explored the possibility of NP playing a regulatory role in this process. We observed that expression of IAV NP in mammalian cells lead to reduced phosphorylation of PKR and its substrate eIF2α. We thus hypothesize that influenza NP is the viral factor that facilitates the inhibition of PKR activation by releasing P58^IPK^ from Hsp40-P58^IPK^ complex. Consistent with this hypothesis, we observed that during IAV infection, the association of NP with Hsp40 coincided with the release of P58^IPK^ from Hsp40. Also, RNAi-mediated inhibition of NP expression in IAV infected cells enhanced the phosphorylation of PKR and its downstream substrate eIF2α. NP inhibition also led to enhanced IRF3 phosphorylation and IFN β production which may be mediated by PKR activation. Collectively, these findings identify a novel role for influenza A virus NP in blocking the PKR-dependent antiviral response in influenza A virus infected cells.

## Results

### Identification of human Hsp40 as an interacting partner of H5N1 IAV NP

A human lung cDNA library was screened using IAV NP as bait, in GAL4 based Matchmaker yeast two-hybrid system (Clontech). Yeast cells (AH-109) were co-transformed with bait and prey plasmids, and selected for growth on selective L^-^T^-^H^-^ plates supplemented with 50 mM aminotriazole. β-galactosidase positive colonies were further analyzed (Fig. S1A). Plasmids from positive colonies were isolated and subjected to DNA sequencing followed by BLAST analysis to identify their cDNA insert. The mammalian chaperone heat shock protein 40 (Hsp40/DNAJB11) was thus identified as an interacting partner of NP. The strength of the NP-Hsp40 interaction was determined using a quantitative β-galactosidase assay, and was found to be comparable to the positive control used in the assay (Fig. S1B, bars 6 and 7 respectively, p-value = 0.0668).

### Transiently expressed IAV NP interacts with Hsp40 in mammalian cells

**Figure 1:**
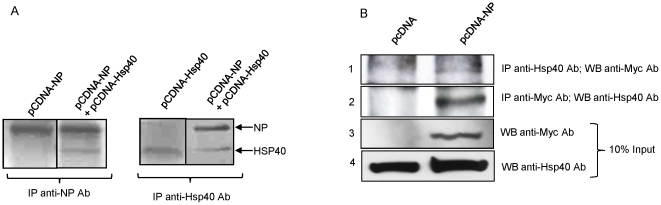
Detection of IAV NP-Hsp40 interaction in mammalian cells tranfected with NP expressing plasmid by co-immunoprecipitation. ***A.*** HEK293T cells were pcDNA3.1-NP and pcDNA3.1-Hsp40 plasmids alone or in combination, followed by metabolic labeling with S^35^. 48 hours post-transfection cells were harvested and IP was setup using anti-NP-specific antibody and anti-Hsp40-specific antibody followed by autoradiography. Lanes 2 and 4 show co-IP of Hsp40 with NP and vice-versa. Lanes 1 and 3 show anti-NP and anti-Hsp40 antibodies were not cross reacting with Hsp40 and NP, respectively. ***B.*** A549 cells were transfected with pcDNA3.1-NP plasmid or control pcDNA3.1 plasmid. Cells were harvested 48 hours post-transfection and immunoprecipitation was setup using anti-Myc tag antibody and anti-Hsp40 antibody, followed by western blotting. Lane 2 of panel 1 shows co-IP of NP with Hsp40 and lane 2 of panel 2 shows co-IP Hsp40 with NP. Lane 1 of panel 1 and 2 represents control samples transfected with empty vector. Panels 3 and 4 show expression levels of NP and Hsp40 in cell lysates.

The NP-Hsp40 interaction in mammalian cells was ascertained using co-transfection of plasmids coding H5N1 NP and Hsp40 in the HEK293T cells. Transfected cells were metabolically labeled with S^35^, and co-immunoprecipitation was performed using the lysates with either NP- or Hsp40- specific antibodies. These results showed that NP co-precipitated with Hsp40 and vice-versa (Fig. 1A, lane 2 and 4). These results were confirmed using A549 lung epithelial cells, which were transfected with NP expressing plasmid, followed by immunoprecipitation. It was observed that ectopically expressed NP could immunoprecipitate endogenous cellular Hsp40 and vice-versa (Fig. 1B, panel 1 and 2). A direct interaction between NP-Hsp40 was further confirmed using a co-immunoprecipitation assay in which ^35^S labeled NP and Hsp40 proteins were expressed from plasmids using *in-vitro* coupled transcription-translation rabbit reticulocyte lysate system (TNT, Promega, Inc) (data not shown). Collectively, these results showed that IAV NP directly interacts with Hsp40.

### IAV NP interacts with Hsp40 in human lung epithelial cells infected with different pandemic and seasonal influenza A viruses

**Figure 2:**
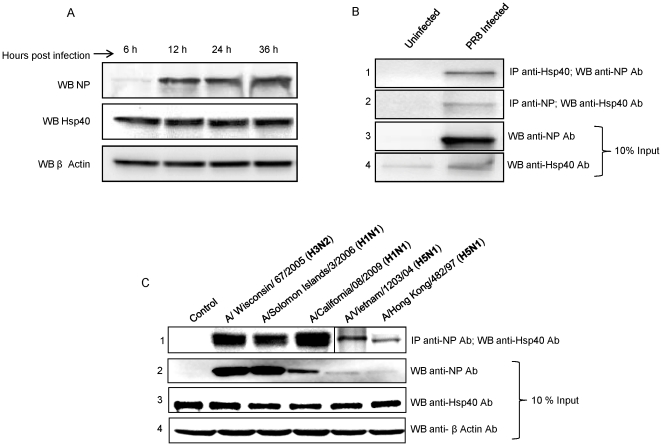
Confirmation of Hsp40 interaction with NP of various influenza A viruses by co-immunoprecipitation. ***A.*** A time course expression analysis was done to check levels of Hsp40 and NP in A549 cells infected with PR8 IAV at 1 MOI. Panel 1 shows NP reached its maximum levels at 12 hours post-infection. Panel 2 shows that Hsp40 levels remained unchanged through the course of infection. ***B.*** A549 cells were infected with PR8 IAV at 1 MOI and harvested 24 hours post-infection. IP was setup using NP-specific antibody and Hsp40-specific antibody. Lane 2 of panel 1 shows co-IP of NP with Hsp40 and lane 2 of panel 2 shows co-IP Hsp40 with NP. Lanes 1 of panel 1 and 2 represents control uninfected samples. Panels 3 and 4 show expression levels of NP and Hsp40 in cell lysates, as detected by western blotting. ***C.*** A549 cells were infected with different influenza A virus isolates, as indicated at 1 MOI. Cells were harvested 24 hours post-infection and IP was setup using NP specific antibody, followed by western blotting. Panel 1 shows co-IP of Hsp40 with NP, panels 2, 3 and 4 show levels of NP, Hsp40 and β-Actin in the cell lysates used for IP. Lane 1 of all panels show control uninfected samples.

To validate the interaction between IAV-NP and Hsp40 in virus-infected cells, we investigated the kinetics of expression of NP and Hsp40 in A549 cells. A549 cells were infected with a laboratory-adapted IAV, A/Puerto Rico/8/1934/A (H1N1) influenza virus (PR8) at a multiplicity of infection (MOI) of 1. Although NP expression was maximal at 24 h post-infection, Hsp40 expression levels remained unchanged throughout the course of infection (Fig. 2A). Therefore, in subsequent experiments, A549 cells were infected with PR8 at an MOI of 1 for 24 h and lysates were prepared. A co-immunoprecipitation assay was performed using infected and control cell extracts. PR8 NP was able to co-precipitate endogenous Hsp40 (Fig. 2B, panel 2). Conversely, Hsp40 was able to co-precipitate NP (Fig. 2B, panel 1). The NP-Hsp40 interaction was also observed when A549 cells were infected with influenza virus isolates belonging to various subtypes (Table 1). Co-immunoprecipitation of proteins from A549 cells infected with these select viral isolates showed that NP co-precipitated with Hsp40 in all cases without exception (Fig. 2C, panel 1). A phylogenetic analysis of influenza NP genes used in the experiment was constructed using the Neighbor-Joining method, nucleotide model Tamura-Nei, in MEGA version 4 (Fig. S2). The diversity of changes in NP amino-acid sequences of the strains used in this study are shown in Fig. S3. The NP genes of IAV used in the study had amino-acid sequence divergence in the range of 1% to 10% from Hatay/H5N1/2004 isolate. These results clearly indicated that the NP-Hsp40 interaction was conserved among seasonal human, avian H5N1 and the 2009 H1N1 pandemic influenza A viruses.

**Table 1:** Influenza A virus strains used in the study.

Lane no.	Strain used for infection	Genbank ID
1	Control Sham treated	_
2	Influenza A/ Wisconsin/ 67/2005 (**H3N2**) (Seasonal Flu)	GenBank: EU097866.1
3	Influenza A/Solomon Islands/3/2006 (**H1N1**) (Seasonal Flu)	NP Sequence unavailable
4	Influenza A/California/08/2009 (**H1N1**) (Pandemic Flu)	GenBank:FJ984366.1
5	Influenza A/Vietnam/1203/04 (**H5N1**)	NP Sequence unavailable
6	Influenza A/Hong Kong/482/97 (**H5N1**)	NP Sequence unavailable

### IAV NP and Hsp40 co-localize primarily in the nucleus of mammalian cells

**Figure 3:**
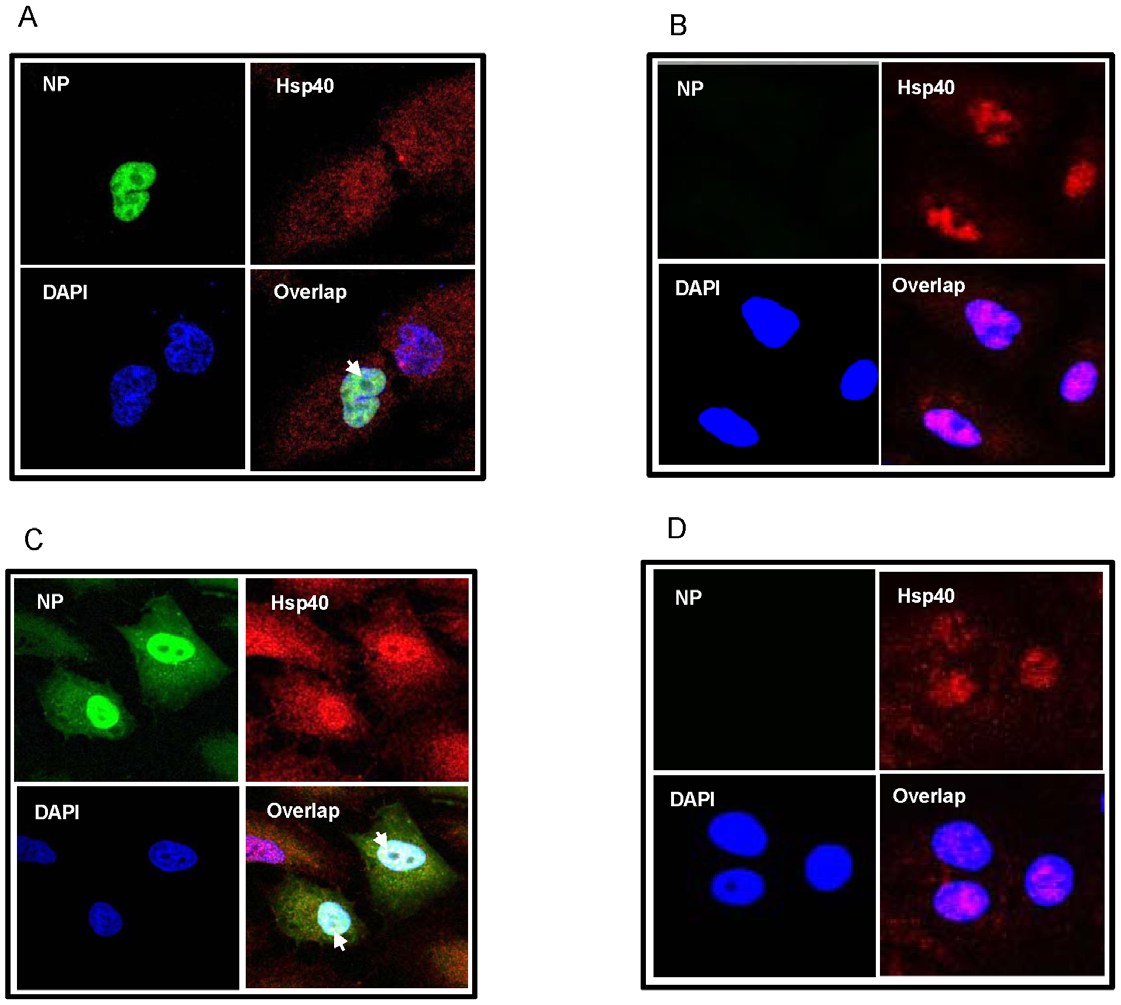
Co-localization of IAV NP and Hsp40 in nucleus of mammalian cells. ***A and B.*** A549 cells were transfected with pcDNA3.1-NP or control pcDNA3.1 plasmid for 24 hours, and cells were fixed and processed for immunostaining. NP was stained using anti-Myc tag specific primary antibody and Alexa488 conjugated secondary antibody (Green). Hsp40 was stained using Hsp40 specific primary antibody and Alexa 594 conjugated secondary antibody (Red). Nuclei were stained with DAPI. ***A*** shows pcDNA3.1-NP transfected cells whereas ***B*** shows control pcDNA3.1 transfected cells. Panels are labeled for their respective staining. Lower right panel shows nuclear colocalization of NP and Hsp40. ***C and D.*** A549 cells were infected with PR8 influenza A virus at 1 MOI for 24 hours, and cells were fixed and processed for immunostaining. NP was stained using anti-NP monoclonal primary antibody and Alexa488 conjugated secondary antibody (Green). Hsp40 was stained using Hsp40 specific primary antibody and Alexa 594 conjugated secondary antibody (Red). Nuclei were stained with DAPI. Panels are labeled for their respective staining. ***C*** shows PR8 infected cells whereas ***D*** shows control uninfected cells. Lower right panel in ***C*** shows primarily nuclear colocalization of NP and Hsp40.

It is known that under stress conditions the expression level of Hsp40 is enhanced and its cellular localization changes from cytoplasmic to nuclear [38], however its distribution in influenza virus infected cells was not studied. Thus we investigated the cellular localization pattern of IAV NP in context to Hsp40 in mammalian cells. We transfected A549 cells with IAV NP expressing plasmid for 24 h, and an immunofluorescence staining was performed with specific antibodies. Results showed that NP and cellular Hsp40 colocalize primarily in the nucleus (Fig. 3A, Lower right panel). Similar results were obtained with A549 cells infected with PR8 virus. Confocal microscopy revealed that NP and Hsp40 were present primarily in the nucleus. However there was significant amount of NP present in the cytoplasm at 24 h post-infection (Fig. 3C, Lower right panel). We also observed that Hsp40 cellular levels were elevated after IAV infection, as compared to uninfected cells (Fig. 3 C and D, upper right panels).

### NP expression and association with Hsp40 coincides with Hsp40-P58^IPK^ dissociation and downregulation of PKR and eIF2α phosphorylation

**Figure 4:**
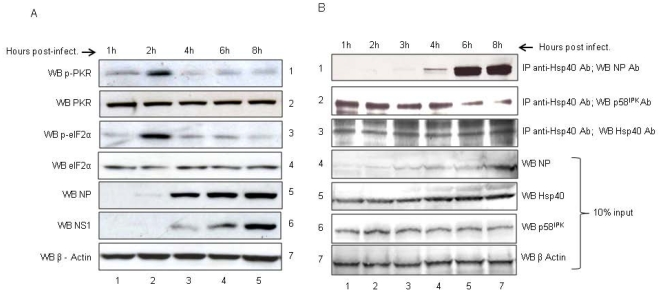
NP expression in IAV infected cells coincides with p-PKR and p-eIF2α downregulation and P58^IPK^-Hsp40 dissociation. ***A.*** A549 cells were infected with PR8 IAV at 1 MOI and cells were harvested at indicated time points post-infection. Western blotting was done to check the expression levels of PKR, p-PKR, eIF2α, p-eIF2α, NP and NS1. Panel 7 shows equal loading trough β-Actin control. This experiment was repeated thrice with similar results. ***B.*** A549 cells were infected with PR8 IAV at 1 MOI and cells were harvested at indicated time points. Protein concentration was measured by Bradford method and equal amount of protein sample from each time point was used to set up IP with anti-Hsp40 antibody. Panel 1 shows the amount of NP co-immunoprecipitated with Hsp40. Panel 2 shows amount of P58^IPK^ co-immunoprecipitated with Hsp40. Panel 3 shows amount of Hsp40 immunoprecipitated at different time points. Lanes 4, 5, and 6 show the levels of NP, Hsp40 and P58^IPK^ respectively, in cell lysates at the indicated time points as detected by western blotting using antibodies specific to NP, Hsp40 and P58^IPK^. Panel 7 shows equal loading trough β-Actin control.

Hsp40 is known to negatively regulate eIF2α phosphorylation through PKR (27). Therefore, we next assessed if changes in PKR and eIF2α phosphorylation occurred during the course of IAV infection of A549 cells. We found that the phosphorylation of both PKR and eIF2α increased initially between 1–2 h post-infection and subsequently declined between 4–8 h post-infection (Fig. 4A, panel 1,3). However the total PKR and eIF2α levels remained unchanged (Fig. 4A, panel 2,4). Interestingly, NP expression also was detected around 4 h post-infection and increased up to 8 h post-infection (Fig. 4A, panel 5) which coincided with a decline in p-PKR and p-eIF2α levels.

During IAV infection P58^IPK^ activity was increased as it was released from Hsp40 binding [27]. We hypothesized that NP might disrupt the P58^IPK^-Hsp40 complex too and liberate P58^IPK^. To investigate this possibility, we monitored changes in the cellular levels of P58^IPK^ and NP associated with Hsp40 during IAV infection. A549 cells were infected with the PR8 virus, harvested at different time points after infection and immunoprecipitation was conducted using equal amounts of total protein and anti-Hsp40 antibody. Western blot analysis revealed that between 4 and 8 h post-infection, the levels of NP associated with Hsp40 continued to rise (Fig. 4B, panel 1) with a concomitant decline in P58^IPK^ associated with Hsp40 (Fig. 4B, panel 2). During this period, total amounts of Hsp40 and P58^IPK^ remained constant (Fig. 4B, panel 5, 6). These results were consistent with the hypothesis of replacement of P58^IPK^ from Hsp40, by NP. These findings indicate that the dissociation of P58^IPK^-Hsp40 complex occurs around 4 to 8 h post-infection, and is associated with downregulation of eIF2α phosphorylation. Taken together, these results suggest that during IAV infection, NP induces the dissociation of the P58^IPK^-Hsp40 complex leading to an inhibition of PKR activation and downregulation of eIF2α phosphorylation.

### IAV NP expression leads to an inhibition of PKR activity and downregulation of eIF2α phosphorylation

**Figure 5:**
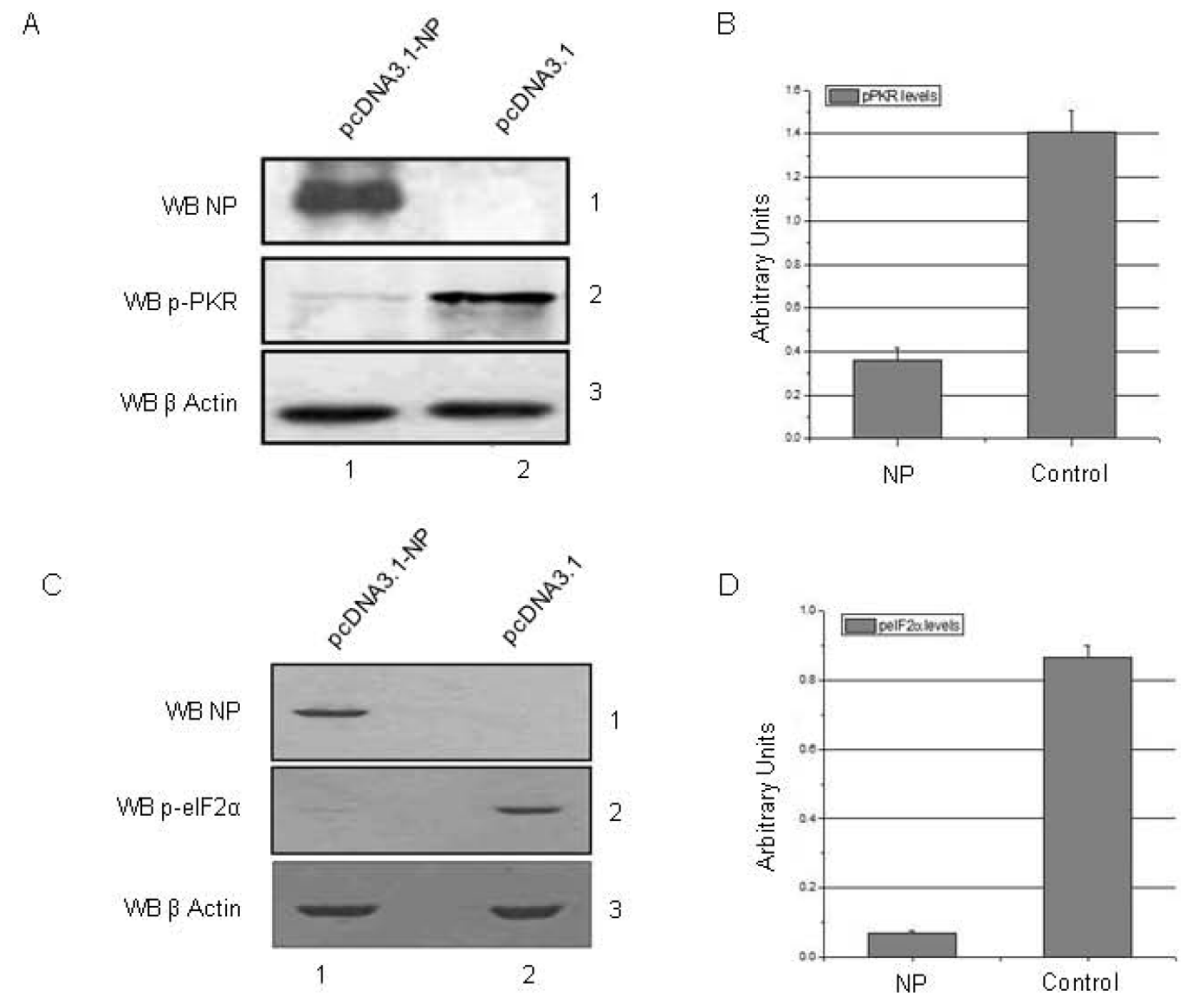
NP expression in mammalian cells leads to downreglation of PKR and eIF2α phosphorylation. HEK293T cells were transfected with NP expressing plasmid (pcDNA3.1-NP) or control plasmid (pcDNA3.1). Cells were harvested at 36 hours post-transfection and cell lysates were subjected to western blotting analysis. ***A.*** Lane 1 of panel 2 shows significant downregulation of pPKR levels in NP transfected cells. Panel 1 shows NP expression level, whereas panel 3 shows equal loading trough β-Actin control. ***B.*** Figure shows graphical representation of relative pPKR levels as measured by western blotting followed by densitometric measurement in 3 independent experiments. Error bars represent standard deviation. ***C.*** Lane 1 of panel 2 shows significant downregulation of p-eIF2α levels in NP transfected cells. Panel 1 shows NP expression level, whereas panel 3 shows equal loading trough β-Actin control. ***D.*** Figure shows graphical representation of relative p-eIF2α levels as measured by western blotting followed by densitometric measurement in 3 independent experiments. Error bars represent standard deviation.

During IAV infection, the NS1 protein inhibits PKR activation by directly interacting with it, and thereby ensuring continued viral mRNA translation [21], [22]. Results from our study indicated that NP may also play a role in inhibiting PKR activity by intercepting this pathway at the level of Hsp40. To examine this aspect, we performed a time course study in HEK293T cells transfected with the plasmid expressing IAV H5N1 NP by monitoring p-eIF2α and p-PKR levels. Western blot analysis of transfected cell lysate showed downregulation of both PKR and eIF2α phosphorylation, which began as early as 12 h and was most prominent at 36 h post-transfection (Fig. S4 A, B). The 36 h time-point was selected to confirm this action of NP, and Western blot analysis results from 3 independent experiments showed significant reduction in the levels of p-PKR (Fig. 5A, panel 2 and Fig. 5B) and p-eIF2α (Fig. 5C, panel 2 and Fig. 5D). To confirm the PKR inhibitory action of NP, we transfected HEK293T cells with NP-GFP expressing plasmid and inhibited NP expression using specific siRNA (Table 2) against it (Fig. S5A). Inhibition of NP expression led to an increase in p-PKR and p-eIF2α levels as compared to control NP-GFP transfected cells (Fig. S5B). These results suggest that the expression of NP leads to inhibition of PKR activity in a NS1 independent manner. This action of IAV NP is likely to be mediated through its interaction with Hsp40.

**Table 2:** Sequences of siRNAs used in the study.

NPsiRNA1	5′AAGCAGGGUAGAUAAUCACUU3′5′GUGAUUAUCUACCCUGCUUUU3′
NPsiRNA2	5′GCAGGGUAGAUAAUCACUCUU3′5′GAGUGAUUAUCUACCCUGCUU3′
NPsiRNA3	5′UCACUCACUGAGUGACAUCUU3′5′GAUGUCACUCAGUGAGUGAUU3′
NS1siRNA1	5′AAGCAGGGUGACAAAGACAUU3′5′UGUCUUUGUCACCCUGCUUUU3′
NS1siRNA2	5′GCAGGGUGACAAAGACAUAUU3′5′UAUGUCUUUGUCACCCUGCUU3′
NS1siRNA3	5′AGACAUAAUGGAUCCAAACUU3′5′GUUUGGAUCCAUUAUGUCUUU3′
NP-GFP siRNA	5′GAGCAGAAAUCCAGGGAAUUU3′5′AUUCCCUGGAUUUCUGCUCUU3′

### siRNA mediated inhibition of NP expression in IAV infected cells, leads to the upregulation of PKR, eIF2α and IRF3 phosphorylation

**Figure 6:**
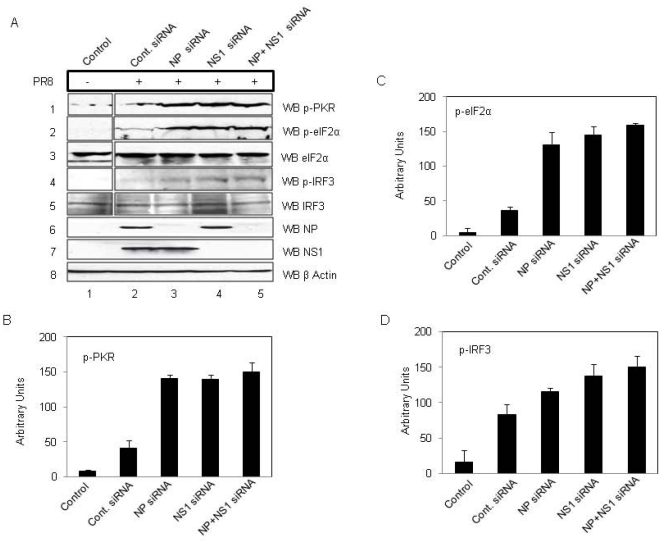
Inhibition of NP expression IAV infected cells upregulates phosphorylation of PKR, eIF2α and IRF3. A549 cells were treated with siRNA against NP, NS1 or NP andNS1 or control siRNA for 6 hours. Cells were then infected with PR8 virus at an MOI of 1. Cells were harvested at 24 hours post-infection and equal amounts of protein from control and treated cell extracts were subjected to western blot analysis. ***A.*** Lane 1 of panels 1, 2 and 4 show control levels of p-PKR, p-eIF2α and p-IRF3, and lane 2 shows the effect of control siRNA on the same. Lane 3 in panel 1, 2 and 4 shows levels of p-PKR, p-eIF2α and p-IRF3 levels in case of NP inhibition. Lane 4 shows effect of NS1 inhibition. Lane 5 shows a synergistic effect of NP+NS1 silencing on p-PKR, p-eIF2α and p-IRF3 levels. Lanes 3 and 5 of panel 6 show the silencing efficacy of NP siRNA and lanes 4 and 5 of panel 7 shows the silencing efficacy of NS1 siRNA. Panel 8 shows equal loading through β-Actin control. ***B, C and D.*** Graphs shows relative p-PKR, p-eIF2α and p-IRF3 levels in virus infected cells pretreated with siRNAs, as measured by western blot followed by densitometric analysis of the protein bands. Plots represent the mean and standard deviation of of three independent experiments.

We suppressed NP expression PR8 IAV infected A549 cells, using a pool of gene specific siRNAs (Table 2), and determined its effect on PKR activity. Expression of NS1 was also blocked using siRNA against NS1 alone and in combination with NP siRNA to establish their exclusive and combined contribution to PKR-inhibition. A549 cells were first transfected with the indicated siRNAs, and 6 hours later were infected with PR8 influenza virus. Infected cells were harvested 24 h post-infection and cell lysates were subjected to western blot analysis. We observed that inhibition of NP expression led to upregulation of PKR phosphorylation, as compared to the control (Fig. 6A, panel 1, Fig. 6B). Increased PKR activity resulted in enhanced phosphorylation of eIF2α and IRF3 (Fig. 6A, panel 2, 4, Fig. 6C, D). Inhibition of NS1 had a similar effect, whereas inhibition of both NP and NS1 had a cumulative effect on upregulation of PKR, eIF2α, and IRF3 phosphorylation (Fig. 6B, C, D).

### Inhibition of NP expression during IAV infection leads to enhanced IFNβ production and reduced virus replication

**Figure 7:**
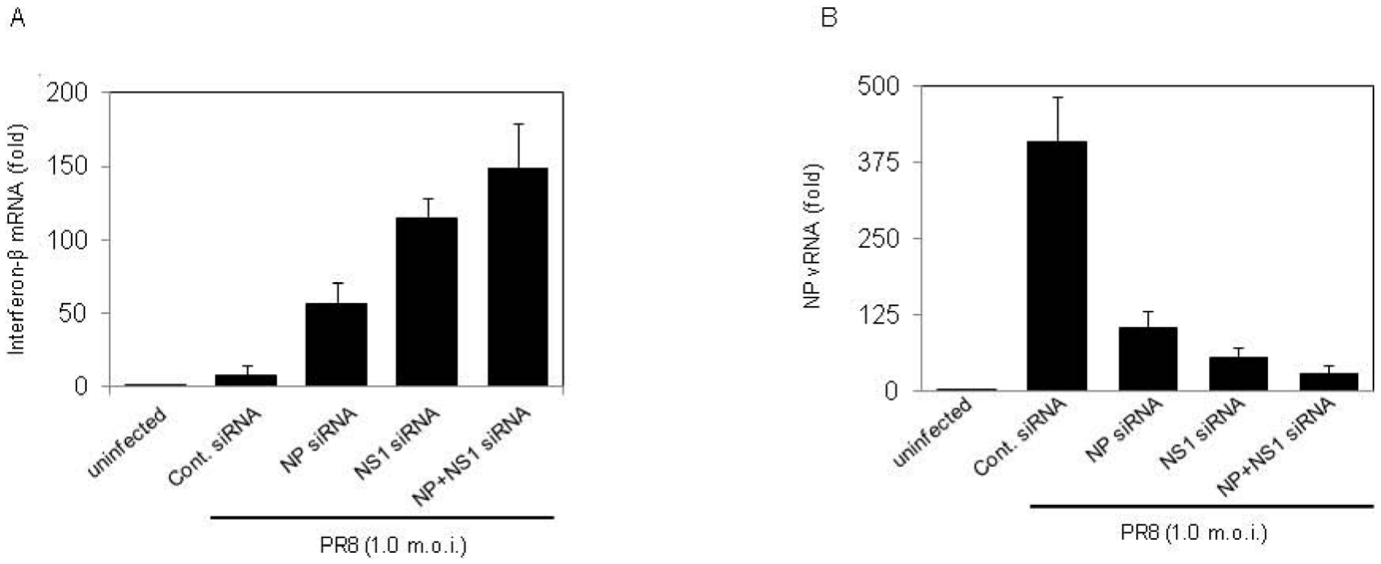
Inhibition of NP expression in IAV infected cells leads to reduced IFNβ production and virus replication. A549 cells were treated with siRNA against NP, NS1 or NP and NS1 together or control siRNA for 6 hours. Cells were then infected with PR8 virus at an MOI of 1. Cells were harvested at 24 hours post-infection, RNA was isolated, cDNA was synthesized and real-time PCR was set up using primer specific for IFNβ and influenza NP vRNA. mRNA levels were normalized against β actin, and the result from triplicate experiments were plotted with standard deviation. ***A.*** Graph shows the effect of NP and/or NS1 inhibition on IFNβ production. ***B.*** Graph shows the effect of NP and/or NS1 silencing on NP vRNA production, which is a measure of virus replication.

PKR mediated activation of IRF3 should lead to an increased IFN response. Previous results confirmed the involvement of NP in the inhibition of PKR and IRF3. To check the further downstream effect of NP, we suppressed the expression of NP using siRNAs as mentioned earlier. 24 hours post-infection, the cells were harvested and RNA was isolated to determine IFNβ and viral RNA levels by real-time PCR using gene-specific primers. The inhibition of NP expression led to increased IFNβ production as compared to control (Fig. 7A, bar 2, 3). Furthermore, the inhibition of NS1 had greater impact on IFNβ production as compared to that of NP, and there was a synergistic effect when both NS1 and NP were inhibited (Fig. 7A, bar 4, 5). Increased IFNβ production should lead to reduced virus replication and reduced production of Influenza vRNA. To confirm this, influenza vRNA levels in the above mentioned samples were measured by real-time PCR. Inhibition of NP or NS1 led to reduced vRNA production as compared to control (Fig. 7A, bar 2, 3, 4), and inhibition of both NP and NS1 together had greater synergistic effect (Fig. 7B, bar 5).

## Discussion

Heat shock proteins are stress response factors which also regulate several cellular processes [39]. The Hsp40 family chaperones are known to play important roles in protein folding, translocation, cell signaling and apoptosis [40]–[42]. Very often they are targeted by viral components for successful virus replication. For example, Hsp40 is known to interact with HIV type 2 Vpx protein and facilitate nuclear import of the pre-integration complex [43]. HIV type 1 Nef protein interacts with Hsp40 to enhance viral gene expression [44]. Hsp40 is also known to interact with the HBV core protein and affect viral turnover [45]. Heat shock proteins are known to affect the viral replication of influenza viruses also. For example Hsp90 is known to interact with influenza virus polymerase components and aid in viral RNA synthesis [46]. Hsp70 is also known to be involved in the nuclear export of the RNP complex and play a role in temperature dependence of IAV replication [47], [48]. Likewise, Hsp40 is also known to regulate PKR signaling in influenza virus infected cells [25]. Similarly, the IAV NP is also a multifunctional protein that interacts with a wide variety of viral and cellular macromolecules, including RNA, PB1 and PB2 subunits of the viral RNA-dependent RNA polymerase and the viral matrix protein [30]–[33]. It also binds to several host factors which include CRM1, UAP56, Alpha-importin 1 and NF90 [33]–[37]. Through these interactions, IAV-NP is known to encapsidate the viral genome, regulate virus transcription and replication, contribute towards pathogenicity of virus, and help in interspecies transmission of the virus [30]. However, so far IAV NP is not reported to play any role in modulating the host antiviral response.

A key component of mammalian antiviral response mechanism is dsRNA dependent protein kinase PKR, which is activated by viral dsRNA [8]. Upon activation, PKR gets dimerized and autophosphorylated at multiple serine and threonine residues. Activated PKR phosphorylates eukaryotic translation initiation factor eIF2α, which in phosphorylated state cannot participate in mRNA translation [12]. This is an important strategy of the host to arrest translation of viral mRNAs thereby limiting viral replication [9], [13]. Another crucial host pathway which is activated by PKR is IRF3-mediated IFNβ production. Activation of PKR is known to enhance IRF3 phosphorylation and nuclear movement where it drives expression of Interferon β production and built up of antiviral host response [14]. Similarly, PKR also has other substrates such as MAPK and iKKß which upon phosphorylation trigger various signaling pathways leading to apoptosis or interferon response [10], [11]. Being such a crucial molecule, PKR is very often the target of viral factors [15]–[18]. In case of influenza virus infection, viral NS1 protein is known to bind directly to PKR and inhibit its activation [20], [21]. NS1 also inhibits the function of retinoic acid inducible gene-I (RIG-I), a cytosolic pathogen sensor involved in the antiviral response [49]. Apart from that, PKR activity is controlled by another mechanism where P58^IPK^, the cellular inhibitor of PKR is activated in influenza virus infected cells [25], [28]. Further, P58^IPK^ itself is inhibited by Hsp40 and is present as P58^IPK^-Hsp40 complex under normal conditions. However upon influenza virus infection, it is released from the Hsp40 complex and inhibits PKR activation [24]. In a recent report, it was shown that M2 protein of influenza A virus stabilizes the P58^IPK^-Hsp40 complex and activates PKR phosphorylation, probably during later stage of infection [50]. However the mechanism of dissociation of Hsp40-P58^IPK^ complex and concomitant PKR inhibition during influenza virus infection remain unknown.

**Figure 8:**
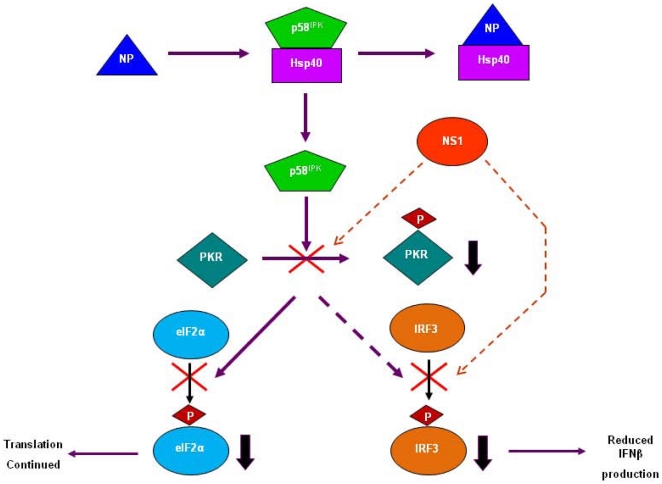
Proposed model for inhibition of PKR mediated host response by influenza nucleoprotein. During influenza virus infection, NP interacts with Hsp40, thereby displacing P58^IPK^ from the Hsp40-P58^IPK^ complex. As a result, there is an increased amount of free P58^IPK^ available in the cell which prevents PKR activation. Downregulation in PKR activity ensures less eIF2α phosphorylation and continued translation from viral mRNAs. On the other hand reduced PKR activity also leads to reduced IRF3 activation and subsequent IFNβ production.

Here, we report that IAV NP interacts with the human chaperone Hsp40 and employs this interaction to mitigate PKR-mediated antiviral response of the host. NP-Hsp40 interaction was identified through a yeast two-hybrid screen and confirmed in a cell-free translation system, in transfected cells and in influenza virus infected cells. The interaction was found to be conserved across different influenza A viruses, ranging from seasonal, avian H5N1 virus and the 2009 H1N1 pandemic virus despite substantial amino acid differences that range from 0–5% within a subtype/group and 6–10% between the subgroups in NP amino-acid sequence. Our findings demonstrate that IAV NP is the viral component that dissociates P58^IPK^ from the P58^IPK^-Hsp40 complex during influenza virus infection in mammalian cells. It was observed that during the course of influenza virus infection in lung epithelial cells, a gradual increase in the association of NP with Hsp40 coincided with a concomitant decrease in P58^IPK^ association with Hsp40. Increased activity of P58^IPK^, promoted by NP, should lead to the inhibition of PKR activation and subsequent downstream effects (Fig. 8). In accordance with the above hypothesis, we observed that ectopic expression of IAV NP in mammalian cells substantially reduced the phosphorylation levels of PKR and eIF2α. Furthermore, siRNA-mediated inhibition of NP expression during influenza virus infection led to increased phosphorylation of PKR and eIF2α, confirming the role of NP in the negative regulation of PKR. Although eIF2α is phosphorylated by other kinases also, namely, HRI, GCN2 and PERK which are activated during stress condition, only PKR is known to be targeted by viral inhibitors [12]. In line with this, NP and NS1 had similar effects on PKR mediated eIF2α phosphorylation; however their synergistic effect was higher than their individual effects (Fig. 6 B).

Activation of PKR signaling during virus infections is known to result in IRF3 phosphorylation and concomitant IFNβ production. However IRF3 is not a direct substrate of PKR and it can get activated by the RIG I pathway, NFκB pathway and other unknown mechanisms [14], [15]. Influenza NS1 protein is known to inhibit PKR, RIG I and NFκB pathways, thus it is expected to have greater impact on IRF3 phosphorylation as compared to NP, which may inhibit only PKR mediated IRF3 phosphorylation [23], [24]. In line with this, we observed that NP inhibition during IAV infection led to enhanced IRF3 phosphorylation, IFNβ production and reduced viral replication; however inhibition of NS1 had greater impact on these events. As expected the synergistic effect of NP and NS1 inhibition on IRF3 activity was higher than their individual effects. The effect of NP on IFNβ production is also reflected on virus replication as siRNA-mediated inhibition of NP led to reduced vRNA production. This effect may also be attributed to the essential requirement of NP for proper functioning of influenza virus polymerase. However the inhibitory action of NP on PKR-mediated host response may also contribute to the reduced virus replication in case of siRNA-mediated inhibition of NP.

Based on our findings, we proposed a model for PKR inhibition by influenza virus nucleoprotein as shown in Fig. 7. According to this model, IAV NP interacts with Hsp40 and facilitates the release of P58^IPK^ from it, which in turn inhibits PKR activation (Fig. 8). Reduced PKR activity, on one hand leads to reduced eIF2α phosphorylation and ensures continued translation from viral mRNAs and on the other hand leads to reduced IRF3 mediated IFNβ production. Therefore, apart from the NS1 protein which is already known to inhibit PKR activation and IRF3 phosphorylation [21], [24], NP also participates in this process, but through a different mechanism involving Hsp40. With structure information of both NP and Hsp40 being available [30], [42], it would be interesting to see which domains and key amino-acid residues are involved in this interaction. Since the NP-Hsp40 interaction is conserved across influenza viruses of various subtypes including the 2009 pandemic H1N1 virus, it serves as an important target for developing anti-viral strategies.

## Materials and Methods

### Yeast Two-Hybrid Screening

GAL4 based Matchmaker (Clontech) yeast two-hybrid system was used for screening human lung cDNA library, as described previously [51]. H5N1 NP (A/Hatay/2004) gene cloned in pGBK vector (Clontech) was used as bait and a mammalian cDNA library cloned in pGAD (Clontech) vector was used as prey. The AH109 strain of yeast was used for co-transformation of bait and prey plasmids. The full-length Hsp40 gene was cloned into pGAD vector and used in yeast two-hybrid assays. Colonies which grew on L^-^T^-^H^-^ plates (Leucine, Tyrosine and Histidine dropout standard dextrose media) supplemented with 50 mM Aminotriazole were considered positive. ß-Gal assays (liquid and filter) were performed as per manufacturer's protocols.

### Cell culture and Plasmids

HEK 293T (human embryonic kidney) and A549 (adenocarcinomic human alveolar basal epithelial) cells were used for transfection and infection experiments respectively. All cells were grown in DMEM medium (Hyclone) supplemented with 10% FCS (Hyclone), 100 units/ml Penicillin Streptomycin solution (Invitrogen). NP gene of A/ Hatay/ 2004 (H5N1) influenza virus was cloned in pCDNA3.1 myc his vector (Invitrogen), pEGFPN1 vector (Clontech) and pGBKT7 vectors (Clontech) to generate myc-tagged, GFP-tagged and GAL4 DNA BD fused NP, respectively. Full-length human Hsp40 gene cloned in the pCDNA3.1 myc his vector was kindly provided by M. D. Amaral (Centri de Genetica Humana Instituto Nacional de Saude Dr. Ricardo Jorge, Lisboa, Portugal).

### Transfection and virus infection assays

All DNA transfections were done using Lipofectamine 2000 (Invitrogen) and cells were maintained in DMEM medium devoid of serum and antibiotics. Six hours post-transfection, culture medium was supplemented with 5% FCS and 24 h post-transfection the medium was replaced with fresh culture medium. All virus infections were done at multiplicity of infection (MOI) of 1 for 1 h in DMEM medium supplemented with 2% BSA (GIBCO). After 1 h incubation, the cells were washed with DMEM once and then grown with DMEM supplemented with 0.2% BSA and 1 µg/ml N-p-tosyl-1-phenyl alanine chloromethyl ketone (TPCK) (Sigma Aldrich). The virus strains used in infection experiments are listed in Table 1.

### Western blotting and antibodies

Cells were lysed using a buffer (20 mM HEPES, pH 7.5, 150 mM NaCl, 1 mM EDTA, 10% glycerol, 1% Triton X-100) supplemented with protease-inhibitors (Roche Diagnostics) and the lysates were subject to SDS PAGE. Anti-NP antibodies were obtained from Abcam and the Immunology and Pathogenesis Branch, Influenza Division, Centres for Disease Control and Prevention, Atlanta, GA, USA. Antibodies against PKR, p-PKR, eIF2α, p-eIF2α, P58^IPK^ and Hsp40 were obtained from Cell Signaling. Anti-ß-actin antibody was purchased from Sigma-Aldrich. Anti-myc tag and anti-NS1 antibodies were purchased from Santa Cruz.

### Immunoprecipitation assays

Cellular lysates were incubated with primary antibody overnight followed by incubation with protein A Dyna beads (Invitrogen) for 2 hours. Beads were washed thrice and the IP products were subjected to Western blotting. NP was immunoprecipitated using anti-NP monoclonal antibody (Immunology and Pathogenesis Branch/IPB, CDC, Atlanta) in case of infection or anti-myc tag antibody in case of transfection. Hsp40 was immunoprecipitated using anti-Hsp40 monoclonal antibody (Cell Signaling).

### Immunofluorescence microscopy

After infection or transfection for 24 h, A549 cells were fixed with 2% paraformaldehyde for 30 min at room temperature. They were permeabilized with 0.5% Triton X-100 for 5 min at room temperature and blocked with PBS containing 2% bovine albumin. Immunostaining was performed using rabbit anti-Hsp40 (Cell Signaling) and mouse anti-NP (IPB, CDC, Atlanta) antibodies. Unbound antibodies were washed away with PBS and cells were incubated with Alexa488 tagged Goat anti-rabbit antibodies and Alexa594 tagged Goat anti-mouse. Nuclei were stained with DAPI. Photomicrographs were captured at 100× magnification using a Leica DM6000B confocal microscope. Images were processed using NIS Elements AR 3.0 software (Nikon).

### RNAi

Control (non-targeting) and NP- and NS1- specific siRNAs of PR8 were purchased from Dharmacon and the cells were transfected using the Dharmafect 1 transfection reagent (Dharmacon). In each case, a pool of three specific siRNAs capable of targeting different regions of NP or NS1 were used (Table 2). A549 cells at a density of 10^6^/well of a 6-well plate were transfected with 90 nM of the indicated siRNA for 6 h prior to infection with A/PR/8/34 at a MOI of 1. Lysates were prepared 24 h post-infection and analyzed for the expression of NP, NS1 and other cellular proteins by Western blotting.

### RNA isolation, cDNA preparation and Real time PCR

Total RNA was isolated from cells using the RNAeasy kit (Qiagen, Valencia, CA, USA) and real-time RT-PCR was conducted using a Stratagene Q3005 PCR machine for expression of IFNβ, β-actin mRNA and NP vRNA. For each sample, 2 µg of RNA was reverse transcribed using Superscript II Reverse Transcriptase (Invitrogen, Carlsbad, CA, USA) according to the manufacturer's directions. Oligo dT primers were used for IFNβ and β-Actin cDNA synthesis. For NP vRNA, cDNA was synthesized as described by Ge *et al* [52]. (Parallel reactions without reverse transcriptase were included as negative controls. Reverse transcription reactions (1/50^th^ of each reaction) were analyzed in using syber green Q-PCR reagents (Stratagene, La Jolla, CA, USA). PCR condition was kept as 94°C for 15 s, annealing at 56°C for 30 s, and extension at 72°C for 30 s for a total of 45 cycles. The threshold cycle number for cDNA was normalized to that of β-actin mRNA, and the resulting value was converted to a linear scale. Data from three independent experiments were taken account for analysis. All data points fell into a normal distribution and there were no outliers. Primer sets used for these studies are provided in Table 3.

**Table 3:** Sequences of primers used for real-time PCR.

β-actin: forward	*5′-* ACC AAC TGG GAC GAC ATG GAG AAA *-3′*
β-actin: reverse	5′- TAG CAC AGC CTG GAT AGC AAC GTA -3′
IFNβ: forward	*5′-* TGG GAG GCT TGA ATA CTG CCT CAA *-3′*
IFNβ: reverse	*5′*- TCT CAT AGA TGG TCA ATG CGG CGT -3′
NP: forward	*5′*- CTCGTCGCTTATGACAAAGAAG-3′
NP: reverse	*5′*- AGATCATCATGTGAGTCAGAC-3′

## Supporting Information

Figure S1 **Human heat shock protein 40 was found to interact with Influenza A nucleoprotein in yeast two-hybrid system**. ***A.*** Yeast two-hybrid screen was performed to find the host interacting partners for H5N1 IAV NP. Results with one of the positive co-transformants (later found to be Hsp40 by BLAST analysis) are shown. Ah109 yeast strain cotransformed with NP-GBK bait plasmid and Hsp40-GAD prey plasmid grew in minimal synthetic YPD media devoid of Leucine, Tryptophan and Histidine amino-acids. Positive colonies grew on plates supplemented with up to 50 mM aminotriazole (AT). A filter β-gal assay was performed to confirm the interaction. Blue colored colonies indicate positive clones. ***B.*** NP-Hsp40 interaction was confirmed by liquid ß-gal assay and was found to be statistically comparable to the positive control used (p-value = 0.0668). In the bar-graph, bar 1 represents untransformed AH109 yeast cells; bars 2 and 3 represent control prey plasmids, bars 4 and 5 represent prey plasmids expressing full-length Hsp40 and NP, respectively; bar 6 represents the co-transformation of Hsp40 and NP plasmids; bar 7 is a positive control (SARS Coronavirus NP both as bait and prey self-associating to form oligomers) [53].
(TIF)

Figure S2 **Phylogenetic analysis of NP sequence used in the study.** A phylogenetic tree was constructed using Neighbor-Joining method, nucleotide model Tamura-Nei, in MEGA version 4 [54]. NP gene sequences from selected human seasonal, avian, swine and 2009 pandemic influenza viral isolates were used. The tree shows evolutionary distances between various strains of influenza. The 2009 pandemic H1N1 NP belongs to the classical swine lineage which had previous limited introductions into humans and is more distantly related to the NP of seasonal or H5 influenza viruses. The H5N1 virus used in this study is shown in red and other IAVs used in infection assays are boxed.
(TIF)

Figure S3 **Amino acid sequence comparison of NP sequence used in the study.** The number and percent difference in amino acids of the IAV subtypes used in the infection assays including seasonal H1N1 and H3N2, avian H5N1 and 2009 H1N1 pandemic are compared to A/*Puerto Rico*/8/1934 (H1N1) virus. Analyses were conducted using the Dayhoff matrix based method in MEGA4 [48].
(TIF)

Figure S4 **NP expression leads to decreased PKR and eIF2α phosphorylation.** ***A and B.*** Time-course analysis of p-PKR and p-eIF2α levels in NP expressing plasmid transfected cells. HEK 293T cells were transfected with pcDNA3.1-NP plasmid and harvested at different time points. Protein amount was estimated by Bradford method and equal amounts of protein from different time points was analyzed on SDS-PAGE and subjected to western blot analysis. Panel 1 of ***A*** *and* ***B*** shows, downregulation of p-PKR and p-eIF2α levels as early as 12 hours (lanes 3 and 4), and was most apparent at 36 hours post-transfection (lanes 7 and 8).
(TIF)

Figure S5 **Inhibition of NP expression leads to increased PKR and eIF2α phosphorylation.** ***A.*** The effect of siRNA-mediated inhibition of NP expression on PKR and eIF2α phosphorylation in NP-transfected HEK293 T cells was checked. Upper panel shows that a 30 nM concentration of siRNA was optimum for silencing. ***B.*** Lower panel shows that when NP expression was silenced, the levels of p-PKR and p-eIF2α went up (lanes 4 and 3) which were otherwise downregulated (lane 1). Lane 2 shows mock transfected cells.
(TIF)
