# Supplementary figures and images for: Influenza A Virus Nucleoprotein Exploits Hsp40 to Inhibit PKR Activation
Source: PLoS One. 2011 Jun 15;6(6):e20215. doi: 10.1371/journal.pone.0020215 (PMC3115951; doi:10.1371/journal.pone.0020215)

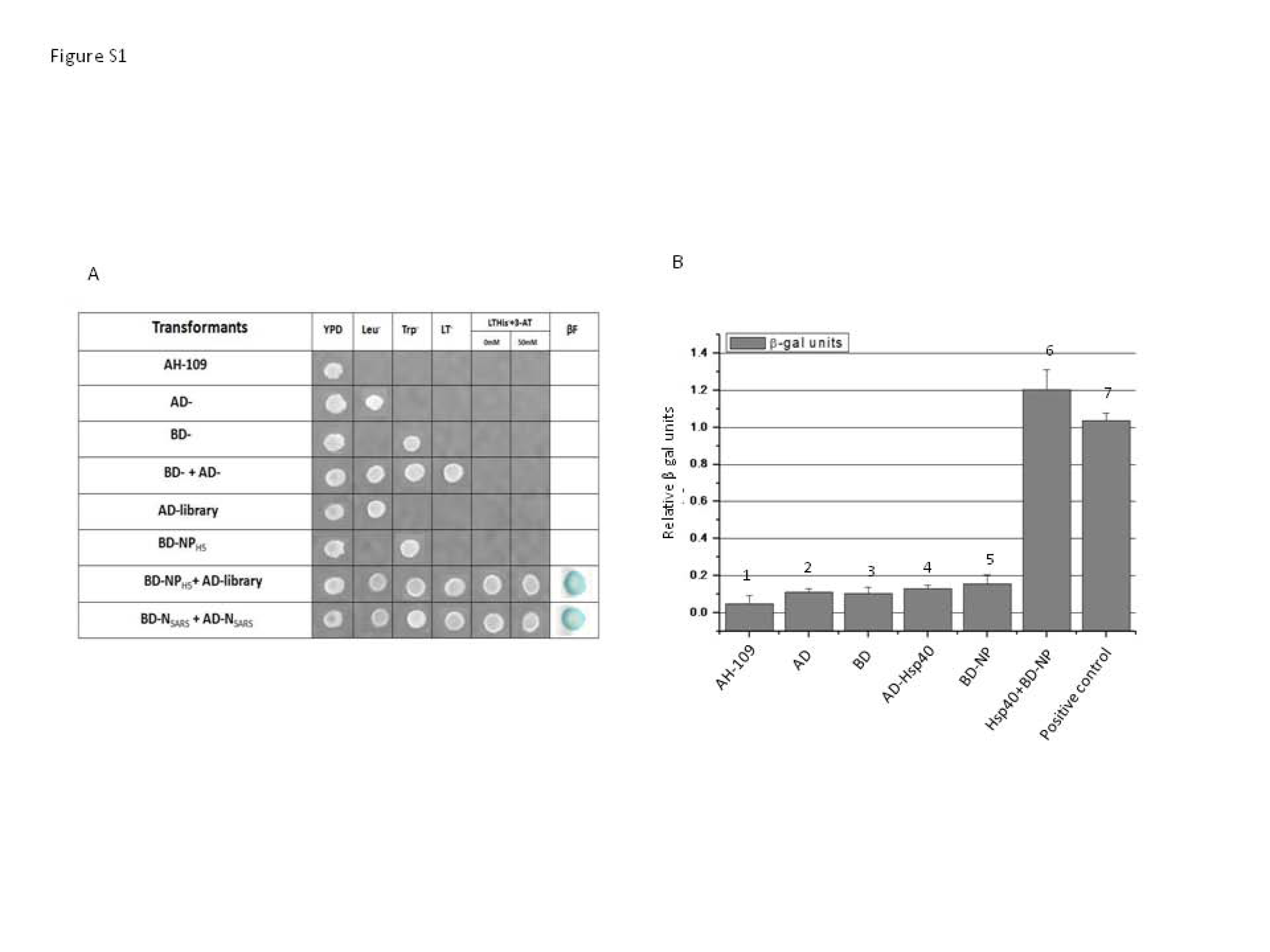

Supplement: Figure S1 — Human heat shock protein 40 was found to interact with Influenza A nucleoprotein in yeast two-hybrid system. A. Yeast two-hybrid screen was performed to find the host interacting partners for H5N1 IAV NP. Results with one of the positive co-transformants (later found to be Hsp40 by BLAST analysis) are shown. Ah109 yeast strain cotransformed with NP-GBK bait plasmid and Hsp40-GAD prey plasmid grew in minimal synthetic YPD media devoid of Leucine, Tryptophan and Histidine amino-acids. Positive colonies grew on plates supplemented with up to 50 mM aminotriazole (AT). A filter β-gal assay was performed to confirm the interaction. Blue colored colonies indicate positive clones. B. NP-Hsp40 interaction was confirmed by liquid ß-gal assay and was found to be statistically comparable to the positive control used (p-value = 0.0668). In the bar-graph, bar 1 represents untransformed AH109 yeast cells; bars 2 and 3 represent control prey plasmids, bars 4 and 5 represent prey plasmids expressing full-length Hsp40 and NP, respectively; bar 6 represents the co-transformation of Hsp40 and NP plasmids; bar 7 is a positive control (SARS Coronavirus NP both as bait and prey self-associating to form oligomers) [53]. (TIF) [file pone.0020215.s001.tif]

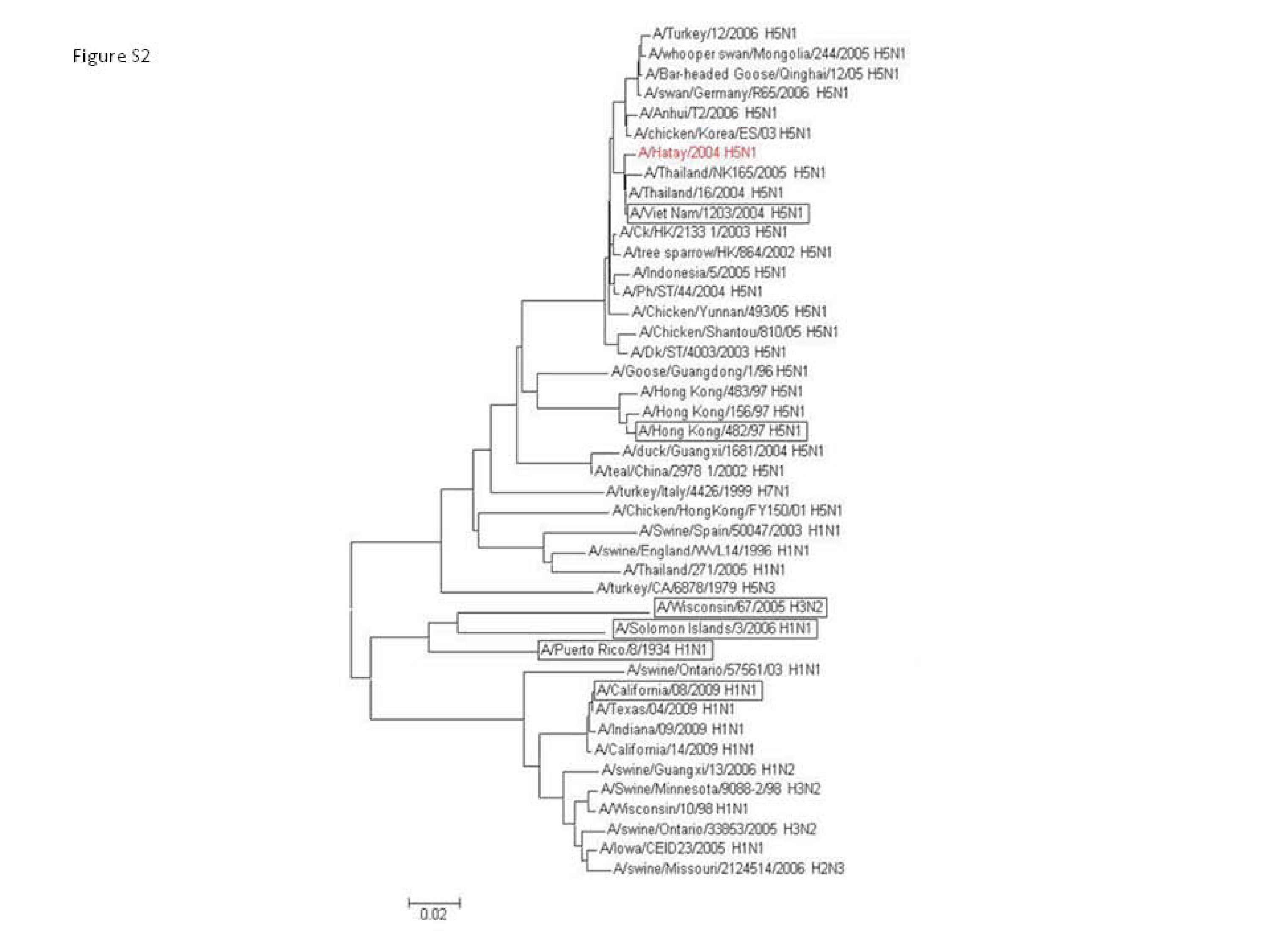

Supplement: Figure S2 — Phylogenetic analysis of NP sequence used in the study. A phylogenetic tree was constructed using Neighbor-Joining method, nucleotide model Tamura-Nei, in MEGA version 4 [54]. NP gene sequences from selected human seasonal, avian, swine and 2009 pandemic influenza viral isolates were used. The tree shows evolutionary distances between various strains of influenza. The 2009 pandemic H1N1 NP belongs to the classical swine lineage which had previous limited introductions into humans and is more distantly related to the NP of seasonal or H5 influenza viruses. The H5N1 virus used in this study is shown in red and other IAVs used in infection assays are boxed. (TIF) [file pone.0020215.s002.tif]

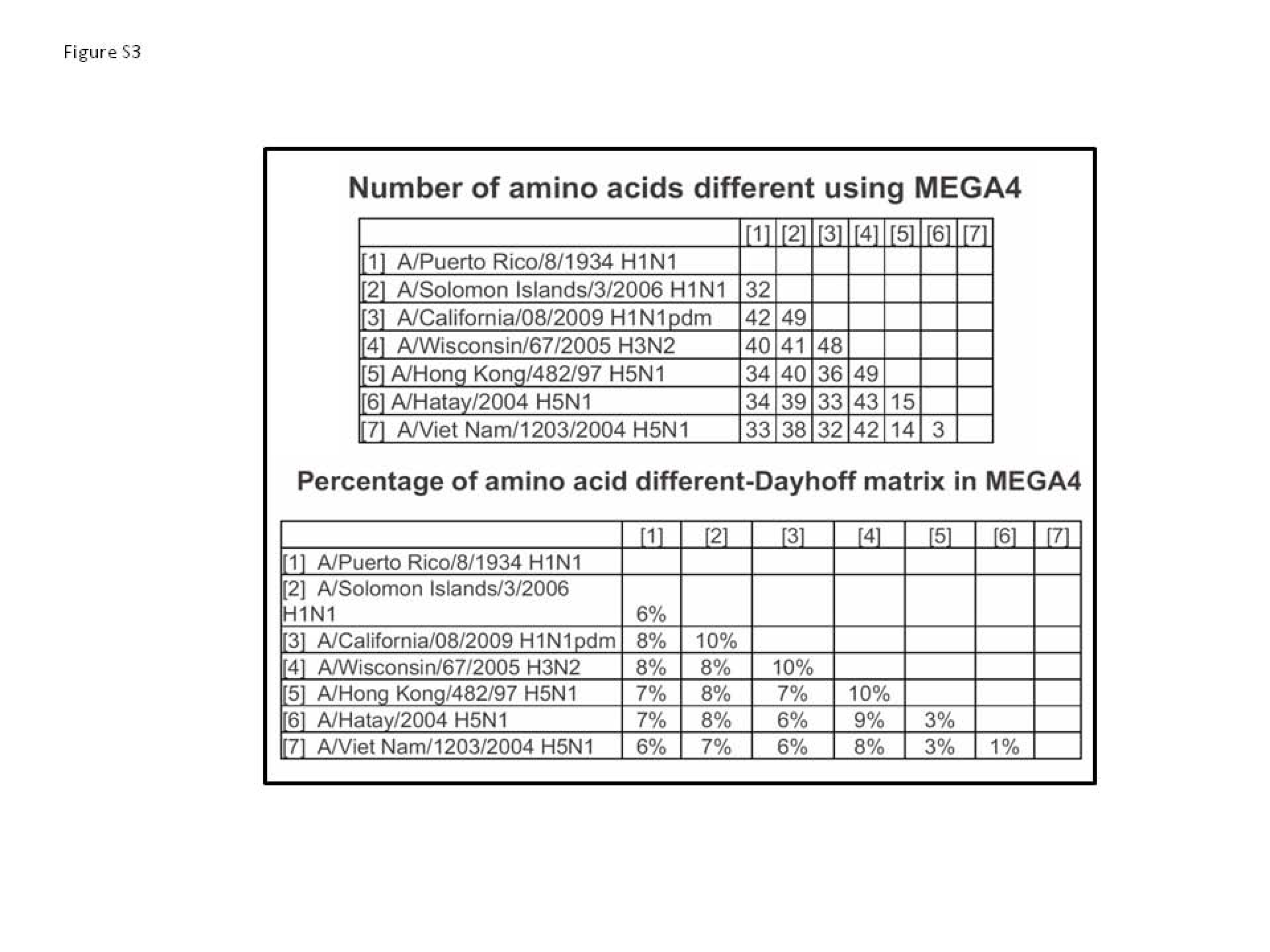

Supplement: Figure S3 — Amino acid sequence comparison of NP sequence used in the study. The number and percent difference in amino acids of the IAV subtypes used in the infection assays including seasonal H1N1 and H3N2, avian H5N1 and 2009 H1N1 pandemic are compared to A/Puerto Rico/8/1934 (H1N1) virus. Analyses were conducted using the Dayhoff matrix based method in MEGA4 [48]. (TIF) [file pone.0020215.s003.tif]

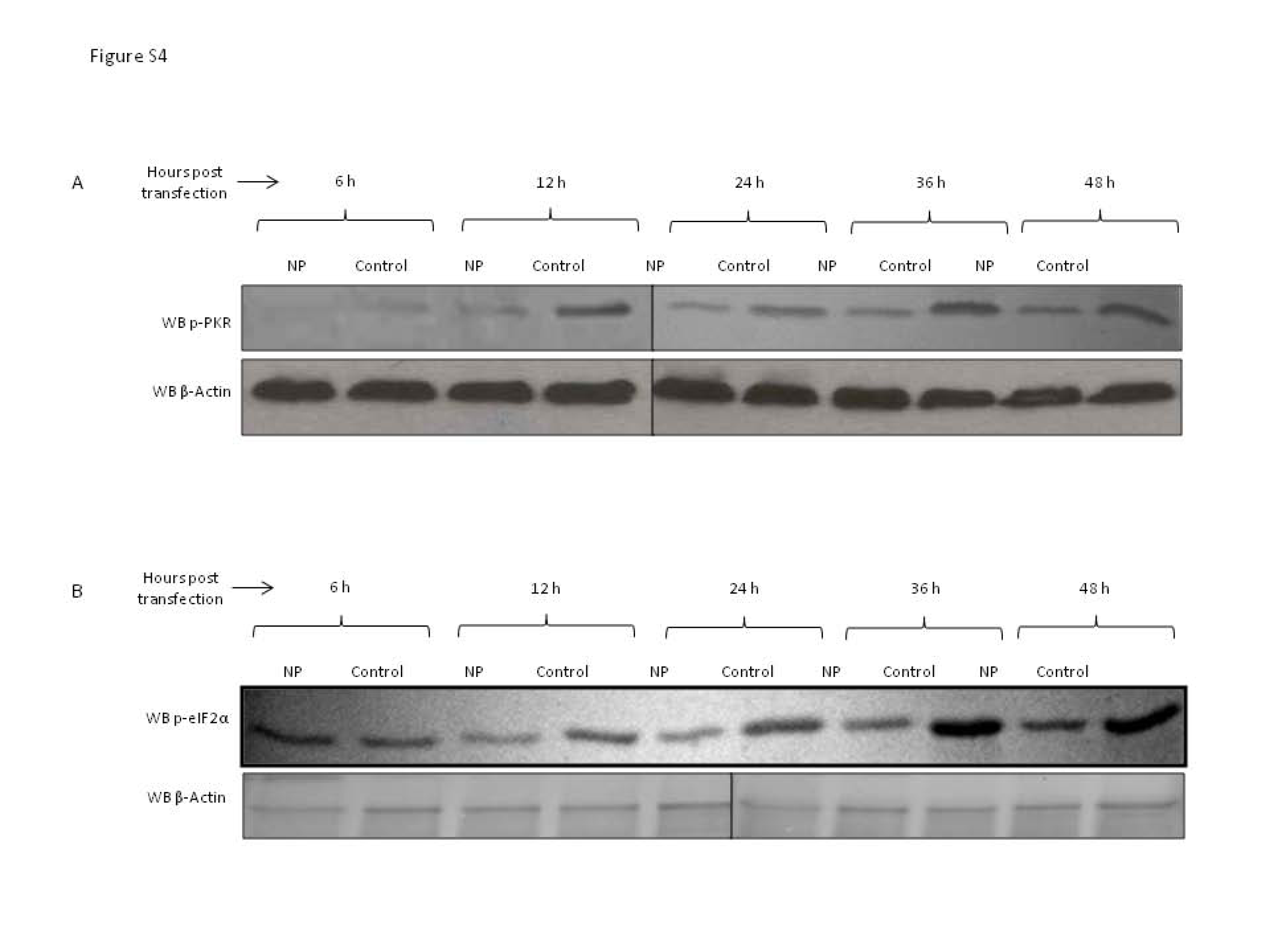

Supplement: Figure S4 — NP expression leads to decreased PKR and eIF2α phosphorylation. A and B. Time-course analysis of p-PKR and p-eIF2α levels in NP expressing plasmid transfected cells. HEK 293T cells were transfected with pcDNA3.1-NP plasmid and harvested at different time points. Protein amount was estimated by Bradford method and equal amounts of protein from different time points was analyzed on SDS-PAGE and subjected to western blot analysis. Panel 1 of A and B shows, downregulation of p-PKR and p-eIF2α levels as early as 12 hours (lanes 3 and 4), and was most apparent at 36 hours post-transfection (lanes 7 and 8). (TIF) [file pone.0020215.s004.tif]

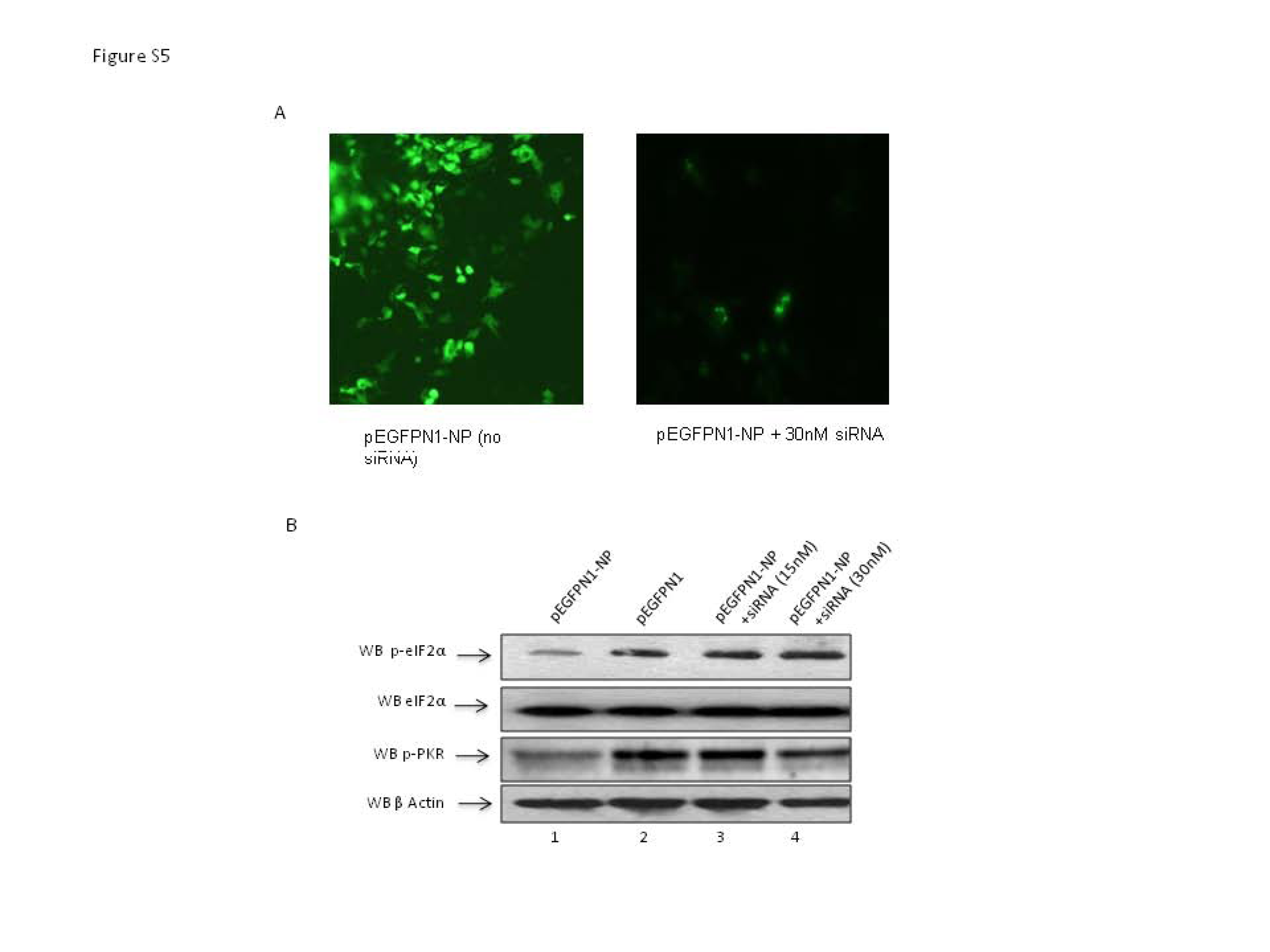

Supplement: Figure S5 — Inhibition of NP expression leads to increased PKR and eIF2α phosphorylation. A. The effect of siRNA-mediated inhibition of NP expression on PKR and eIF2α phosphorylation in NP-transfected HEK293 T cells was checked. Upper panel shows that a 30 nM concentration of siRNA was optimum for silencing. B. Lower panel shows that when NP expression was silenced, the levels of p-PKR and p-eIF2α went up (lanes 4 and 3) which were otherwise downregulated (lane 1). Lane 2 shows mock transfected cells. (TIF) [file pone.0020215.s005.tif]
